# Cardiac troponin T as a serum biomarker of respiratory impairment in amyotrophic lateral sclerosis

**DOI:** 10.1002/acn3.52126

**Published:** 2024-06-25

**Authors:** Teresa Koch, Rachel Fabian, Leonie Weinhold, Franz‐W. Koch, Saman Barakat, Sergio Castro‐Gomez, Torsten Grehl, Sarah Bernsen, Patrick Weydt

**Affiliations:** ^1^ Department of Neuromuscular Diseases, Center for Neurology University Hospital Bonn Bonn 53127 Germany; ^2^ Department for Medical Biometry, Informatics and Epidemiology University Hospital Bonn Bonn 53127 Germany; ^3^ Department of Pulmology Neukölln Hospital Berlin Germany; ^4^ Department of Parkinson, Sleep and Movement Disorders, Center for Neurology University Hospital Bonn Bonn 53127 Germany; ^5^ Institute of Physiology II University Hospital Bonn Bonn 53115 Germany; ^6^ Department of Neurology Alfried ‐ Krupp‐Hospital Essen Germany; ^7^ German Center for Neurodegenerative Diseases (DZNE) Bonn 53127 Germany

## Abstract

**Objective:**

Informative biomarkers are an urgent need in the management of amyotrophic lateral sclerosis. Serum cardiac troponin T is elevated in the majority of amyotrophic lateral sclerosis patients and increases with disease progression. We sought to establish the informative value of cardiac troponin T with regard to respiratory function, a major prognostic factor in amyotrophic lateral sclerosis.

**Methods:**

In this retrospective observation, we analyzed two independent hospital‐based cohorts (*d* = discovery cohort; *v* = validation cohort) regarding serum cardiac troponin T (*n*_*d*_ = 298; *n*_*v*_ = 49), serum neurofilament light chain (*n*_*d*_ = 117; *n*_*v*_ = 17), and respiratory tests (*n*_*d*_ = 93; *n*_*v*_ = 49).

**Results:**

Serum cardiac troponin T, in contrast to serum neurofilament levels, was associated with the respiratory domain of the revised amyotrophic lateral sclerosis functional rating scale and with pulmonary function parameters, namely forced vital capacity % (*r* = −0.45, *p* = 0.001) and slow vital capacity % (*r* = −0.50, *p* = 0.001).

Serum cardiac troponin T reliably discriminated benchmarks of slow vital capacity <80% (AUC 0.73, 95% CI 0.62–0.84) and <50% (AUC 0.80, 95% CI 0.68–0.93), forced vital capacity <80% (AUC 0.72, 95% CI 0.61–0.83) and <50% (AUC 0.79, 95% CI 0.67–0.91).

**Interpretation:**

Our findings position cardiac Troponin T as a valuable serum biomarker in amyotrophic lateral sclerosis, complementing neurofilaments and expanding the understanding of underlying physiological mechanisms. In clinical practice, serum cardiac troponin T can flag benchmarks of compromised respiratory function.

## Introduction

Amyotrophic lateral sclerosis (ALS) is a fatal neurodegenerative disorder characterized by the degeneration of central and peripheral motor neurons. This leads to progressive skeletal muscle weakness and spasticity, culminating in respiratory failure within very few years.^1^ Currently, treatment options for ALS are limited. Therapy development and the challenging disease management requires reliable biomarkers to guide treatment decisions and help define critical disease stages.^2^ Neurofilament light chain (NfL) levels reflect neuroaxonal damage and are readily quantifiable in blood and in cerebrospinal fluid. They support the diagnosis, convey prognostic information and have been accepted as therapy response markers by regulatory authorities.^3^, ^4^, ^5^

Serum cardiac troponin T (cTnT) and I (cTnI) levels are widely used as markers of myocardial injury. Recently, serum cTnT has emerged as a promising candidate serum biomarker for ALS, tracking disease severity and progression.^6^, ^7^ Accumulating evidence suggests that degenerating skeletal muscle is an extracardiac source of serum cTnT, and can result in chronic serum cTnT elevations in neuromuscular disorders independent of myocardial damage.^8^, ^9^, ^10^

Respiratory impairment is one of the main determinants of survival in ALS and may occur at any time during the variable course of the disease, which may initially manifest subtly and unspecific.^11^, ^12^ Early recognition of respiratory dysfunction helps to ensure that noninvasive ventilation (NIV), one of the most life‐prolonging therapies, is discussed in a timely fashion.^13^, ^14^ Moreover, the quantification of the respiratory impairment helps gauge individual prognosis.^15^

The respiratory domain of the ALS functioning rating scale revised (ALSFRSr), while well established as a predictor of survival,^16^ is a comparatively crude three‐item, 12‐point metric of respiratory function. In a clinical setting respiratory function is commonly evaluated with a range of lung parameters that indirectly capture the inspiratory and expiratory muscle functions in pulmonary function testing (PFT). Among these test modalities slow vital capacity (SVC) and forced vital capacity (FVC) are the most widely used in clinical practice and research studies.^14^, ^17^ In ALS, they are recommended to monitor respiratory impairment and provide functional benchmarks for initiating interventions like NIV (SVC or FVC below 80% or 50%), and percutaneous gastrostomy placement (FVC below 50%).^18^, ^19^

The objective of the present study was to investigate the relationship between the functional implications of cTnT serum levels and respiratory dysfunction as captured by ALSFRSr and PFT.

## Materials and Methods

### Study design and patient characteristics

**Figure 1:**
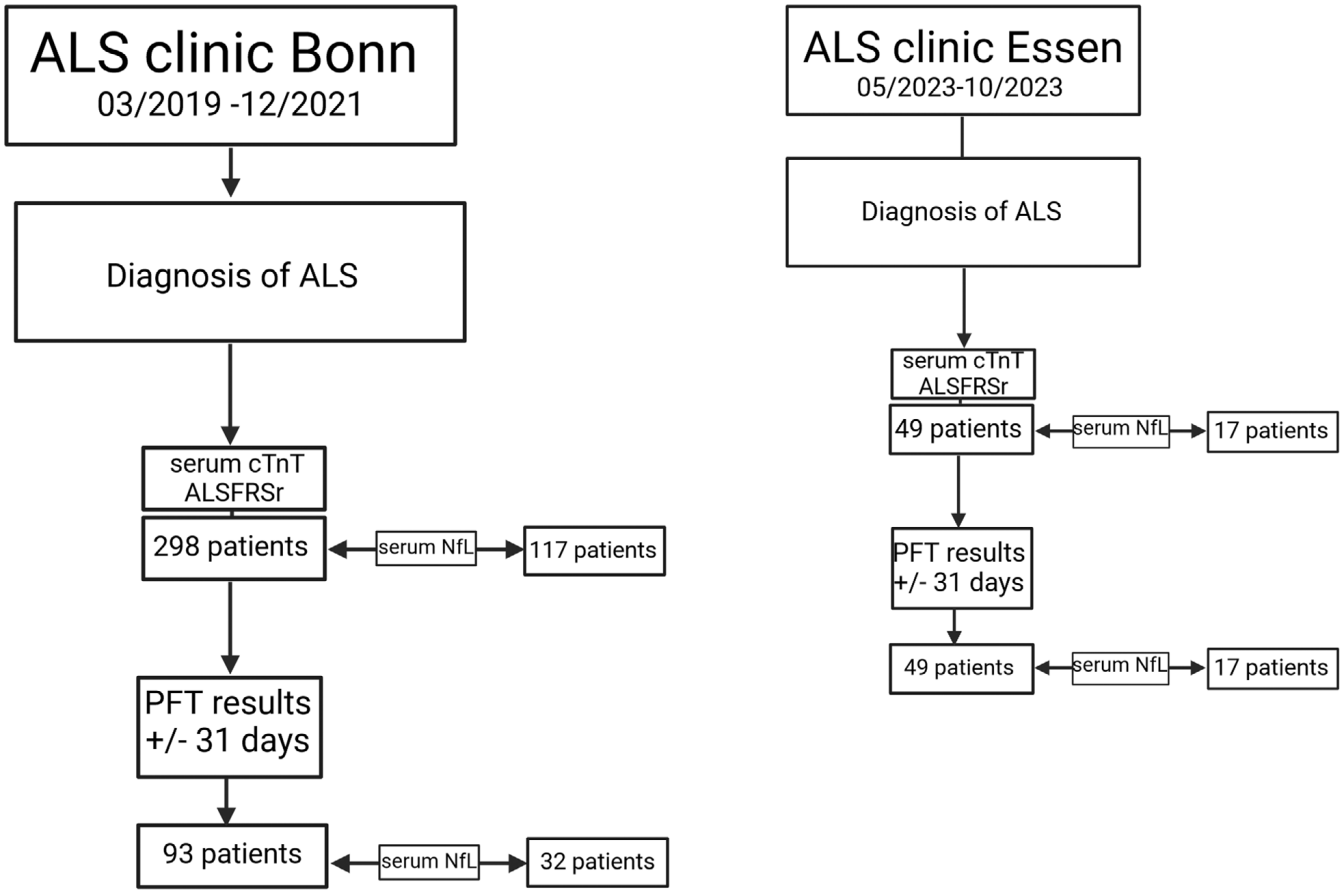
Flow chart of retrospective analysis of two independent study cohorts (discovery cohort Hospital Bonn, 2019–2021; validation cohort Hospital Essen, 2023) was performed targeting the same clinical characteristics: the diagnosis of ALS following the EFNS task force, serum cTnT, serum NfL measurements, and ALSFRSr scale. Results of pulmonary function tests were included when available ±31 days to the latter. ALS, amyotrophic lateral sclerosis; ALSFRSr, revised ALS functional rating scale; cTnT, cardiac troponin T; PFT, pulmonary function test; serum NfL, serum neurofilament light chain.

The present retrospective cross‐sectional study was conducted on two independent hospital‐based cohorts, one from the University Hospital Bonn and one from the Alfried Krupp Hospital Essen (Fig. 1). We first reviewed the files of all patients seen at the ALS clinic of the University Hospital Bonn from 01 March 2019 to 31 December 2021, providing a convenient study size (discovery cohort, *n*_*d*_ = 298). We included patients with a diagnosis of ALS according to the El Escorial diagnostic criteria's categories “possible, probable, and definite”^20^ including progressive muscle atrophy (PMA), determined by board‐certified neurologists (PW, RF, and SB). ALSFRSr, serum cTnT, and serum cTnI are collected as part of the clinical routine at the Bonn clinic.

Serum NfL testing was implemented in routine testing with a lag of several months and thus is only available for a subset of the Bonn patients (*n*_*d*_ = 117). In this cohort, we identified 93 patients with PFT measurements (SVC and FVC) in a time window of ±31 days of the serum cTnT sampling, of which 32 had additional NfL sampling.

Demographic data included sex, age, height, weight, and body mass index (BMI). Disease history information included date and region of ALS symptom onset and disease duration (time interval from symptom onset).

The Essen cohort served as an independent validation cohort (*n*_*v*_ = 49) with data on demographics, disease history, ALSFRSr, serum cTnT, serum NfL, and PFT (SVC).

As the laboratory measurements and clinical assessments were part of the routine clinical work up and retrospectively analyzed, no formal consent was needed per statement of our institutional ethics review board (Ethics Board decision letter 324/20).

### Biomarker measurements

Serum cTnT was measured using the high‐sensitivity electrochemiluminescence immunoassay (ECLIA) in a commercial laboratory (Labor Volkmann Karlsruhe, Germany) as described previously.^6^ Serum NfL was measured in an academic laboratory (University of Ulm, Department of Neurology) with single‐molecule arrays (Simoa, Quanterix Corporation, Lexington, MA, USA) according to the instructions supplied by the manufacturer.

### Respiratory function assessments

PFT readouts were obtained at the Bonn University Pulmonology department with a Vyntus® Body Plethysmograph (Vyaire Medical GmbH Höchberg). In the Essen clinic SVC measurements are part of the routine work up and obtained with a Vitalograph Spitotac MODEL 7000 (Vitalograph GmbH Hamburg).

### Statistical analysis

All statistical analyses were performed with GraphPad Prism® (10) (GraphPad Software Inc., San Diego, USA). Microsoft Excel 2019® was used for data storage (Microsoft Redmond, Washington, USA). Biorender.com was utilized for figures BioRender (Toronto, ON Canada).

Patient characteristics are presented as absolute and relative frequencies for categorical variables and as means with standard deviations (SDs) or median with interquartile ranges (IQR) for continuous variables. For analysis, the biomarker serum cTnT and serum NfL were log‐transformed (natural logarithm) providing normality distribution.

Correlation between cTnT with pulmonary function tests and ALSFRSr were calculated with Pearson's test.

To calculate the diagnostic utility of serum cTnT and NfL for the distinction of clinically relevant thresholds of SVC and FVC (80% and 50%, respectively) the area under the curve (AUC) was calculated with receiver operating characteristic (ROC) curve analyses with 95% confidence intervals (CI). The optimal cutoff points were determined based on the Youden Index.

All analyses were initially conducted using the discovery cohort and significant findings were subsequently tested in the validation cohort. The significance level was set with an alpha level of 0.05 and p‐values were adjusted by the Bonferroni correction for multiple testing.

## Results

### Clinical characteristics

The basic characteristics of the discovery cohort and the validation cohort are described in Table 1. The two cohorts were similar regarding key demographic features such as age, sex ratio, ALSFRSr scale, and proportion of cases with spinal versus bulbar onset. The discovery cohort (Bonn) had a significantly longer median disease duration at sample collection (20 months) than the validation cohort (5 months), reflecting the earlier initiation of the study.

**Table 1:** Baseline characteristics of the discovery and the validation cohort of patients with amyotrophic lateral sclerosis.

	Discovery cohort (*n*_*d*_ = 298)	Validation cohort (*n*_*v*_ = 49)
Sex, female, *n* (%)	133 (44.6%)	30 (61.2%)
Mean age, years (±SD)	63.9 (± 10.1)	62.5 (± 12.2)
BMI kg/m2 (±SD)	24.2 (± 4.0)	25.5 (6.2)
Disease duration, months, median [IQR]	20 [0–37]	5 [3–7]
Spinal onset, *n* (%)	231 (77.5%)	36 (80%)
ALSFRSr, median [IQR]	34 [26.5–39]	39 [34–45]
ALSFRSr respiratory, median [IQR]	11 [9.3–12.0]	12 [11–12]
ALSFRSr decay rate/month (±SD)	1.02 (± 1.27)	2.58 (± 3.20)
ALSFRSr respiratory decay rate/month (± SD)	0.13 (± 0.30)	0.23 (± 0.62)
SVC %, mean (±SD)	82.1 (± 23.9)	80.6 (± 25.8)
cTnT ng/L, mean (±SD)	30.8 (± 39.2)	27.4 (± 42.3)
cTnT >14 ng/L, *n* (%)	193 (64.8%)	23 (46.9%)
cTnT (ln), mean (±SD)	3.0 (± 0.9)	2.72 (± 0.96)
NfL pg/mL, mean (±SD)	109.9 (± 87.2)	65.8 (± 66.4)
NfL >45 pg/mL, *n* (%)	94 (80.3%)	7 (41.2%)
NfL (ln), mean (±SD)	4.5 (± 0.7)	3.42 (± 1.7)

ALSFRSr, revised amyotrophic lateral sclerosis functional rating scale; BMI, body mass index; cTnT, cardiac troponin T; IQR, interquartile range.; ln, natural logarithm; NfL, neurofilament light chain; SD, standard deviation.

**Figure 2:**
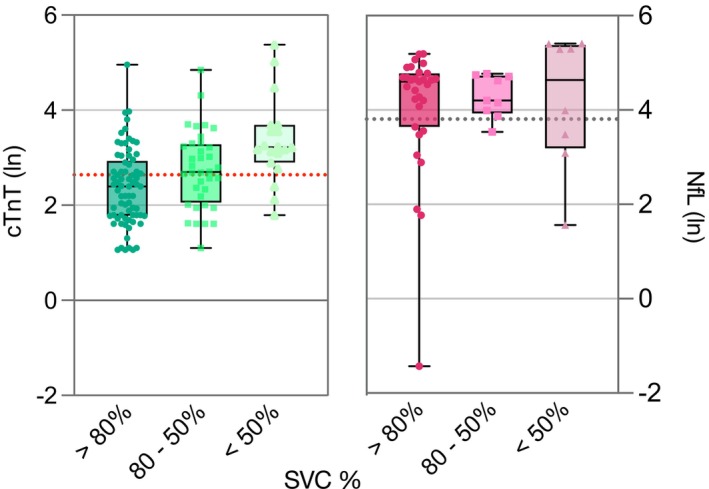
Three groups of SVC % impairment (>80%, 80–50%, and <50%) were formed following clinically important thresholds of respiratory impairment in ALS disease. Box and whiskers plot with minimum and maximum range present the distribution natural logarithm of serum cTnT levels (left) and NfL levels (right) of all patients (discovery and validation cohort) grouped by respiratory stages. The dashed horizontal red line (left) demonstrates the cTnT cutoff value of 14 ng/L (2.64 ln) for myocardial damage. The dashed horizontal black line (right) is the cutoff value of NfL at 45 pg/mL (3.81 ln). cTnT, cardiac troponin T; ln, natural logarithm; NfL, neurofilament light chain.

The mean of serum cTnT levels in the discovery cohort was 30.8 ng/L (± 39.2), that is, >2 times above the upper reference limit (URL) of 14.0 ng/L. In total, 193 (65%) of 298 patients had elevated serum cTnT. None of these patients had elevated serum cTnI levels, ruling out cardiac involvement. In the validation cohort 24 of measurements (47%) were elevated with a similar mean of 27.4 ng/L (±42.3). This is in line with previous reports.^7^, ^21^ The mean serum NfL was 109.9 pg/mL (±87.2) and elevated in 94 patients (80.3%) of 117 patients in the discovery cohort. Similarly, the mean NfL serum level of the validation cohort was elevated above URL at 65.80 pg/mL (±66.4), albeit in a smaller proportion of cases (41.2%). The analysis of both serum biomarker elevations in relation to stages of respiratory impairment, categorized by SVC as >80%, 50–80%, and <50%, revealed an increase of average serum cTnT with decreasing SVC category. However, such a trend was not observed for NfL levels (Fig. 2).

### Correlations with clinical parameters and pulmonary function

**Figure 3:**
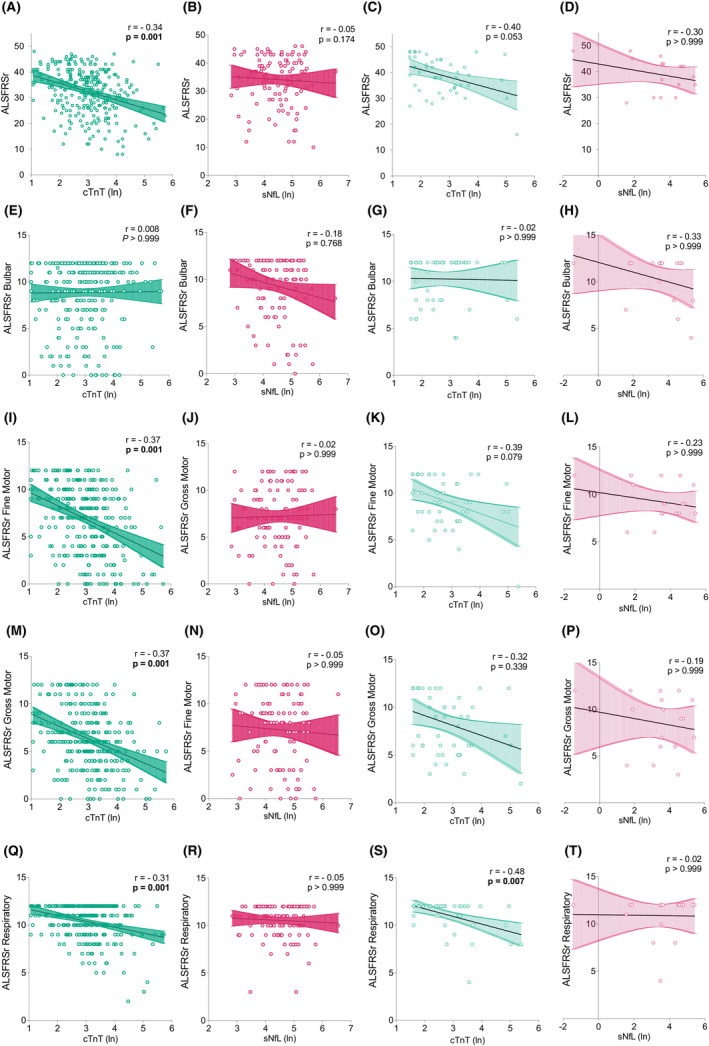
Serum cTnT and NfL levels in correlation with ALSFRSr in two cohorts of ALS patients. Colored areas represent 95% CI. The graphs show correlations of cTnT (green) and of NfL (pink) with the ALSFRSr scale and its four domains (bulbar, fine motor, gross motor, and respiratory). The first two columns demonstrate correlations of serum biomarkers cTnT and NfL with the ALSFRSr domains in the discovery cohort (*n*_*d*_ = 298) and the second two columns in the validation cohort (*n*_*v*_ = 49). ALSFRSr, revised ALS functional rating scale.

**Figure 4:**
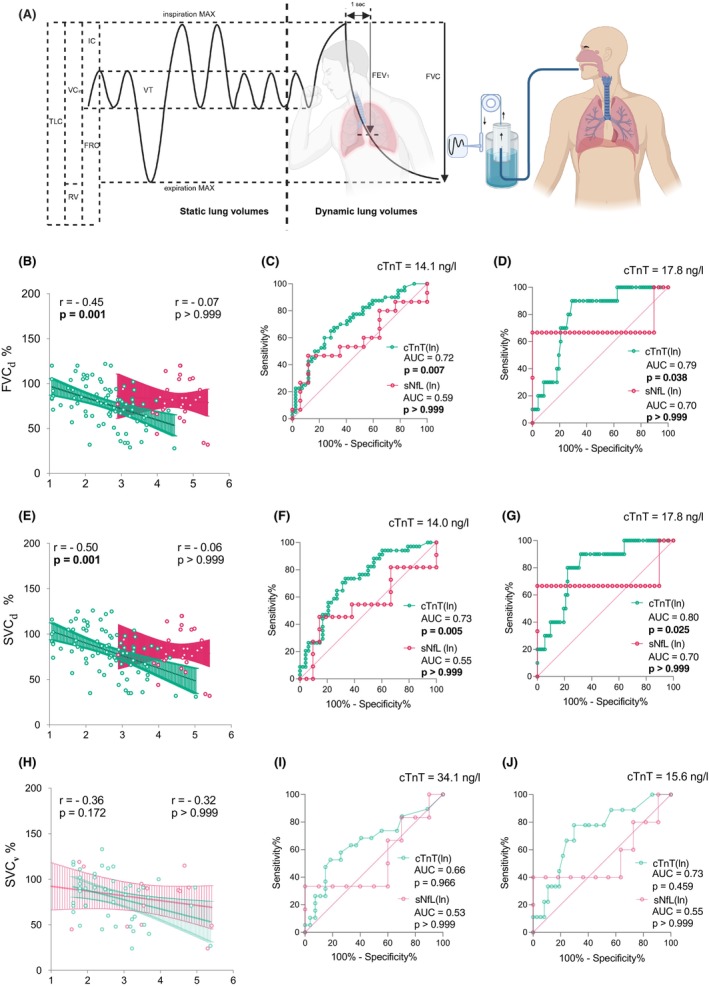
The relationship of serum cTnT and NfL with respiratory impairment measured by pulmonary function tests. Upper Figure (A): the schematic panel shows and the principle of pulmonary function testing (PFT) with static and dynamic lung volumes. Below: Correlation plots between natural logarithm of serum cardiac troponin T (green) and serum neurofilament light chain (pink) with FVC% (B) and SVC% (E) in the discovery cohort and with SVC% (H) in the validation cohort. Colored areas represent 95% confidence interval (CI). Receiver operating characteristic (ROC) curve analyses were performed for cTnT ln and NfL ln distinguishing FVC 80% (C), FVC 50% (D) and SVC 80% (F), SVC 50% (G) in the discovery cohort, and SVC 80% (I), SVC 50% (J) in the validation cohort. The appropriate serum cTnT levels were calculated with Youden analysis, presented in ng/L in every ROC plot. AUC, area under the curve; cTnT, cardiac troponin T; FEV1%, forced expiration in first second; FRC, function residual capacity; FVC%, forced vital capacity; IC, inspiratory capacity; RV, residual volume; SVC, slow vital capacity; TLC, total lung capacity; VC, vital capacity; VT, tidal volume.

Serum cTnT levels correlated strongly and negatively with the total ALSFRSr (*r* = −0.34, *p* = 0.001), and – pertinent to the present study – with its respiratory domain (*r* = −0.31, *p* = 0.001) (Fig. 3). This correlation was fully recapitulated in the smaller validation cohort (*r* = −0.48, *p* = 0.007). The serum NfL levels, in sharp contrast, showed no correlation with the respiratory domain. As respiratory function is one of the most important prognostic factors in ALS,^15^, ^22^ we were compelled to further probe the relationship of serum cTnT with respiration. We found that serum cTnT showed significant negative correlations with the selected parameters, namely FVC (−0.45, *p* = 0.001) and SVC (−0.50, *p* = 0.001) (Fig. 4). While the correlation with SVC was consistent with the validation cohort (*r* = −0.36, *p* = 0.172), statistical significance was likely limited by sample size. To test the diagnostic potential of serum cTnT levels to flag PFT declines below clinically important thresholds, we generated ROC curves (Fig. 4). The most robust results were observed for SVC below 80% (AUC = 0.73, 95% CI 0.62–0.84) and below 50% (AUC 0.80, 95% CI 0.68–0.93), and FVC below 80% (AUC 0.72, 95% CI 0.61–0.83) and 50%. (AUC 0.79, 95% CI 0.67–0.91). The optimal cutoff values for serum cTnT to detect impaired respiration measured as FVC, and SVC below 80% or 50% ranged in between 14.0 and 22.4 ng/L.

## Discussion

Our data offer a novel interpretation of cTnT as a serum biomarker for respiratory dysfunction in amyotrophic lateral sclerosis, an issue that had been addressed by previous studies with limited success.^23^, ^24^ Our key finding is the robust correlation between serum cTnT with PFT and the ALSFRSr respiratory domain, supporting the concept that respiratory skeletal muscles drive, or at least substantially contribute to the serum cTnT level elevations in ALS. Particularly noteworthy is the strong correlation with SVC, the most accurate PFT parameter to capture respiratory impairment in ALS patients.^25^, ^26^

These results are in line with our and other previous studies reporting the relationship of serum cTnT with skeletal muscle function measured by ALSFRSr.^6^, ^7^ We confirm the high prevalence (62%) of serum cTnT elevations above the URL and independent of cTnI, arguing for a noncardiac origin of cTnT.^6^, ^7^, ^9^, ^10^, ^27^, ^28^

Our findings hold the potential to immediately affect clinical practice for several reasons: Serum cTnT levels are useful in discriminating relevant respiratory thresholds (SVC and FVC 80% and 50%, respectively) recommended for informing decisions on important interventions (NIV implementation, indication for PEG). Of note, this is especially true for patient subpopulations where pulmonary functional assessment is particularly unreliable, namely ALS patients with bulbar symptoms and/or with frontotemporal dementia (ALS/FTD).^7^, ^16^

In our dataset, serum cTnT is already altered in patients with mildly impaired respiratory function, even before the ALSFRSr respiratory domain is affected. Thus, serum cTnT levels could prove useful to uncover early stages of respiratory insufficiency. In support of this interpretation, earlier case reports already documented longitudinal measurements of serum cTnT tracking progressive respiratory failure and changes in PaO_2_/CO_2_.^29^, ^30^ Another study reported that increased serum cTnT levels were associated with more severe respiratory involvement.^21^ To externally validate the presented relationship of serum cTnT and respiratory impairment, future studies should examine serum cTnT in relation to the current benchmarks symptoms such as orthopnea, unrefreshing sleep, daytime somnolence, and any evidence of nocturnal desaturation.^12^

Further research is also necessary to see longitudinal measures and Kaplan–Meier analyses of survival against cTnT, NfL, and PFT. Of particular interest might be the biomarker response to therapeutic interventions, including NIV, but also treatment with tofersen. We believe that a key strength of our approach lies in the repurposing of serum cTnT, an established and readily available biomarker.

It is well established that neurofilament levels reflect the rate of neuroaxonal damage.^31^

NfL have excellent diagnostic and prognostic performance in ALS, but our data show that they are not correlated to respiratory function. This contrast underscores that serum cTnT captures an aspect of the disease that is missed by neurofilament levels (either in serum or in cerebrospinal fluid).

A limitation is the observational nature of our investigation. We sought to mitigate this by including a discovery and a (albeit small and not perfectly matched) validation cohort from an independent ALS center in Essen, Germany.

Of note, the Essen ALS center is a secondary care facility, which likely contributes to the remarkably short median diagnostic delay of 5 months. In contrast, the discovery cohort was from Bonn University Hospital, a large tertiary care center.

Also, hospital cohorts, while by nature not rigorously stratified or otherwise standardized, provided comparably robust findings. However, the use of a time window of ±31 days for inclusion of PFT data might have introduced a sampling bias potentially overrepresenting patients at a certain stage of their disease progression.

While the validation cohort does not fully confirm all correlations found in the discovery cohort, possibly because of lack of power, the overall agreement between the two cohorts strongly argues for the robustness of the observed correlation between serum cTnT and respiratory function. Although the correlation analyses between serum cTnT and respiratory function parameters yielded statistically significant results, correlations alone cannot establish causality. Further research is clearly necessary to increase the understanding of underlying pathophysiology of the skeletal muscle in ALS patients, and in particular, the respiratory muscles, including the diaphragm. Future studies might correlate serum cTnT with data of diaphragmatic ultrasound or response on phrenic nerve stimulation or ultrasound.

## Conclusion

We conclude that serum cTnT levels can be repurposed as a biomarker in ALS allowing remarkably robust conclusions on the respiratory capacity of ALS patients. This novel insight could support therapeutic decision‐making. We outline serum cTnT as a biomarker complementing neurofilaments by covering a different disease aspect that could aid with prognosis and future management of the disease. It will be critical to determine how these two serum markers perform in combination.

## Author Contributions

T.K., S.B., S.C‐G., and P.W contributed to the conception and design of the study. T.K., P.W., T.G., F.W.K., L.W., R.F., S.C‐G., and S.B contributed to the acquisition and analysis of data. T.K., S.B., and P.W contributed to drafting the text or preparing figures.

## Conflict of Interest

The authors have no conflict of interest to declare.

## Data Availability

Anonymized data will be made available upon reasonable requests to the corresponding author. The data are not publicly available due to privacy or ethical restrictions.
